# Heat Stress Prevention in Construction: A Systematic Review and Meta-Analysis of Risk Factors and Control Strategies

**DOI:** 10.3390/ijerph21121681

**Published:** 2024-12-17

**Authors:** Mehdi Torbat Esfahani, Ibukun Awolusi, Yilmaz Hatipkarasulu

**Affiliations:** School of Civil & Environmental Engineering, and Construction Management, The University of Texas at San Antonio, San Antonio, TX 78249, USA; mehdi.torbatesfahani@my.utsa.edu (M.T.E.); yilmaz.karasulu@utsa.edu (Y.H.)

**Keywords:** construction workers, heat-related illness, heat stress, control strategies, risk factors

## Abstract

In hot and humid work environments, construction workers can experience heat stress and heat-related illnesses (HRIs). While several studies have investigated engineering and administrative control methods to prevent certain heat stress risk factors, a comprehensive understanding of all existing risk factors and their corresponding control strategies is still lacking. It is crucial to identify gaps in current control strategies and develop a safety management framework for effective heat stress control by implementing existing measures. In addition, the effectiveness of the most common control strategies must be rigorously evaluated to ensure their efficacy and to guide future research aimed at enhancing these strategies or developing more effective ones. This study employed a mixed literature review methodology to address this knowledge gap. A structured literature review investigated and synthesized heat stress risk factors and control methods to find the gaps in control options to address underestimated risk factors. Furthermore, a comprehensive systematic literature review, including trend analysis, scientometric analysis, and meta-analysis, determined research foci and evaluated the effectiveness of the heat stress control methods. The scientometric analysis identified 11 clusters, encompassing key research themes such as environmental risk factors (e.g., high-temperature environments, climate change), administrative controls (e.g., work–rest schedules, climate change risk assessment), and personal interventions (e.g., cooling vests and sleep-related strategies). These findings highlight that the most commonly studied control methods are cooling vests, work–rest schedules, and cooling interventions. According to these results and the availability of quantitative results, the meta-analysis evaluated nine datasets of reductions in core body temperature by using types of cooling vests and anti-heat-stress uniforms and established the significant effectiveness of this control strategy in mitigating heat stress with a medium effect size. Moreover, five potential research studies have been identified to address gaps in control strategies for certain underestimated risk factors, including leveraging sensor technologies, conducting control training, dynamic work–rest schedules, using cutting-edge PPE, and governmental initiatives. Insights gained from this study enhance decision making for resource allocation, selection of control options, and intervention prioritization within a heat-stress-control framework based on the safety management system. The findings also highlight the effectiveness of cooling vests and areas that need to be developed, and evaluate potential heat-stress-control methods in construction.

## 1. Introduction

The construction industry is acknowledged globally to be significantly impacted by heat-related hazards and illnesses [1]. Construction accidents increase in summer due to the hot and humid environments in construction job sites [2]. In addition, construction workers experience more than a third of fatalities in the United States due to heat stress because most of their activities are conducted outdoors [3]. Heat stress is the accumulation of the heat generated by metabolic, environmental, and worn-clothing factors minus the amount of heat dissipated from the body [4]. Although numerous technological and equipment advances have replaced labor activities, much outdoor work is still manual work, which increases the susceptibility to heat stress [5]. Heat stress is an issue that can lead to outdoor workers engaging in moderate to heavy tasks for extended durations in hot and humid environments, with a lack of access to air cooling, conditioning, and ventilation [6]. This enhances side injuries and accidents and reduces productivity on construction job sites by increasing the possibility of human errors stemming from heat-related illness (HRI) and fatigue [7].

Different research studies have identified risk factors that contribute to heat stress. It is crucial to comprehend these risk factors to control heat stress’s impacts effectively. The crucial factors contributing to the heat hazards are environmental, individual, demographic, and occupational. Environmental factors encompass parameters such as air temperature, humidity levels, heat radiation, and air movement [8,9]. Personal factors consist of physical health and conditions, body mass index (BMI), alcohol or caffeine intake, the effects of clothing, the capacity of the body to acclimate to heat exposure (known as acclimatization), levels of fatigue, risk awareness, knowledge, job proficiency, and physiological elements like heart rate, blood pressure, metabolic equivalents, and hydration status [8,9,10]. Age, ethnicity, vulnerability, type of dwelling, religion, migration status, characteristics of the building environment, etc., are included in demographic factors [11]. Occupational or construction-specific factors involving working times, working hours spent outdoors, job position, workload, work pace, management strategies, work–rest schedule, and type of construction project are the most crucial heat stress risk factors in construction [12,13]. These can all lead to severe HRIs, such as heat stroke, exhaustion, syncope, rash, and cramps, which are among the most critical HRIs. While several studies have addressed these risk factors and related cases, existing research often lacks consolidation, leaving gaps in systematically identifying and organizing these factors and their associated control strategies [13,14]. For instance, studies have highlighted limitations in addressing comprehensive risk categorizations and practical implementations of control methods in construction [3,8].

Various strategies have been explored and examined to prevent these severe HRIs and mitigate heat stress risk factors. First, engineering methods involve using techniques, technologies, and equipment to eliminate heat-related hazards from the construction job site. These include controlling air temperature and increasing airflow through convection, creating barriers between workers and heat radiation, air conditioning and ventilation, and utilizing cooling systems or equipment [14,15]. Additionally, cooling vests and anti-heat-stress uniforms represent technologies to reduce body temperature during recovery or rest periods, protecting the body from heat stress [16,17]. Second, administrative controls are equally important in reducing heat stress among construction workers. These include implementing work–rest schedules, providing health education and training, managing daily workloads and intensity, and offering personal protective equipment (PPE), outdoor shade, and suitable clothing options [9,10]. Finally, self-monitoring of responses or personal controlling options have proven to be an effective method for preventing HRIs. Workers can adopt practices such as taking breaks during work hours, staying hydrated by drinking cold water, taking rest breaks regularly, wearing light and single layers of clothing, and using appropriate PPE [10]. Moreover, studies have indicated that being in a healthy physical condition has the potential to prevent HRIs [18]. Using technologies such as environmental and physiological monitoring sensors can add further control barriers to these strategies and aid in early detection of signs indicating a risk of HRIs [19]. Although numerous studies have assessed methods to prevent specific heat stress risk factors, a comprehensive understanding of all existing risk factors and their corresponding control strategies remains incomplete. Identifying gaps in current control strategies is crucial, especially those that overlook or underestimate certain risk factors. Also, it is essential to establish a safety management framework to implement existing measures effectively. Moreover, the effectiveness of the most common control strategies must be rigorously evaluated to ensure their efficacy and guide future research aimed at improving these strategies or developing more effective ones.

This study conducts a mixed literature review to critically explore, evaluate, and synthesize articles to answer five research questions (RQs). This research effort not only synthesizes and evaluates existing heat stress control methods but also presents future research directions for addressing and mitigating risks that have not been addressed in prior studies. The RQs are defined as follows:

RQ1: What risk factors contribute to heat stress (or HRIs) in construction?

RQ2: What are the heat stress (or HRI) control methods used in construction?

RQ3: What are the research foci on heat stress (or HRI) risk factors and control methods in construction?

RQ4: What is the effectiveness of the most commonly used heat stress (or HRI) control methods in construction?

RQ5: What are the future research directions for heat stress (or HRI) prevention and control in construction?

A structured literature review answers RQ1 and RQ2, while scientometric analysis and meta-analysis are employed to answer RQ3 and RQ4. Unlike existing studies that often address heat stress risk factors or control methods in isolation, this research combines these approaches to provide a comprehensive understanding by categorizing risk factors and systematically evaluating control strategies. Integrating scientometric and meta-analysis methods ensures a data-driven identification of research foci, such as cooling vests, and assesses their effectiveness through pooled evidence. Finally, based on a critical review of the existing literature, RQ5 elaborates on prospective research directions, focusing on emerging technologies, dynamic interventions, and sociocultural considerations areas that remain underexplored in the current body of research.

This study provides valuable insights into improving construction safety practices in addressing essential gaps in understanding heat stress risk factors and control strategies. By categorizing risk factors into eight distinct groups and control methods into three levels, engineering, administrative, and personal, this research offers a structured framework to guide interventions to mitigate heat stress and heat-related illnesses. The findings emphasize actionable solutions, such as the effectiveness of cooling vests and work–rest schedules, which can be implemented to enhance worker safety, productivity, and well-being in extreme environments. This study not only bridges gaps in existing research but also provides construction managers, site supervisors, and policymakers with evidence-based strategies to develop comprehensive heat stress prevention plans tailored to the unique challenges of the construction industry.

## 2. Background

### 2.1. Heat Stress Risk Factors and Heat Indexes

Heat stress is the overall heat burden experienced by a worker who is exposed to metabolic heat, extreme environmental parameters, such as hot and humid environments, and increasing retention of body heat affected by the clothing that is worn [14]. Rowlinson et al. indicate six primary factors that impact heat stress, including air movement and temperature, humidity, radiant heat, clothing, or PPE, effects, which moderate the heat exchange between the body and the environment, and the metabolic heat generated by physical activities [7]. The primary factors, known as environmental factors, were initially formulated in the wet bulb globe temperature (WBGT) and the temperature work limit (TWL) indices [20]. These indices account for multiple factors, such as air temperature [21], humidity [22], wind speed, clothing, and atmosphere pressure [23] to assess heat stress. These indices rely on environmental factors and overlook other crucial parameters, such as personal characteristics and occupational conditions, that significantly influence heat stress.

To fill this gap, several heat stress and heat stress models have been developed, incorporating environmental factors; physiological parameters, such as heart rate, blood pressure, energy consumption, respiratory exchange, fatigue, and oxygen uptake; occupational factors, including working activities and time; and other individual factors such as health conditions, the proportion of body fat, body mass index (BMI), drinking/smoking habits, etc. [8,23,24,25,26].

In addition to the environmental, physiological, and occupational factors identified in existing heat stress models, other significant factors have been identified in the literature as contributing to the complexity of heat stress risk. Demographic factors, such as age, gender, and ethnicity, can increase vulnerability, particularly for older workers and expatriates who may be less acclimatized to extreme heat [1,9]. Cultural and societal norms also play a critical role, with practices such as wearing restrictive clothing for religious or cultural reasons exacerbating heat strain by impeding the body’s natural cooling processes [27]. Furthermore, personal factors, including hydration status, sleep patterns, and past medical history, significantly affect individual susceptibility to heat-related illnesses [5,8]. Organizational factors, such as inadequate workplace safety cultures, insufficient cooling and hydration strategies, and lack of proper training programs, have been identified as barriers to effective heat stress management, especially when production and safety concerns are not adequately balanced [9,28]. Moreover, PPE-related factors, such as poorly designed protective equipment restricting ventilation or sweat evaporation, exacerbate heat strain for workers in high-risk environments [7,29]. Societal factors, such as geographic susceptibility and income levels, have also been shown to affect how workers respond to heat stress, particularly in low- and middle-income countries where access to cooling systems and healthcare may be limited [27]. Lastly, while policies and regulations exist to mitigate heat stress, their effectiveness is often undermined by inconsistent enforcement and a lack of proactive monitoring, highlighting a need for more robust regulatory frameworks [30]. Synthesizing these factors will provide a more comprehensive understanding of the multifaceted nature of heat stress and the need for integrated approaches that consider a broad range of hazard dimensions.

### 2.2. Heat-Related Illnesses and Strategies to Control Them

Extreme temperatures and heat stress can increase the possibility of skin cancer and injury rates [3,31], sleeplessness, and sunburn [32], and reduce productivity [33,34] and workers’ performance [2]. Moreover, HRIs are the most terrible consequences of heat stress in construction. Heat stroke, rhabdomyolysis, exhaustion, cramps, syncope, and rashes are the most significant HRIs in construction. Thus, it is necessary to devise more effective strategies to prevent HRIs, thereby decreasing the mortality rate among construction workers [35,36]. Control strategies are crucial and classified into engineering, administration or management, and individual categories.

Engineering control methods encompass strategies such as implementing heat alert programs on work sites through the integration of GIS and BIM or establishing smart construction sites that incorporate wearable sensing devices capable of monitoring physiological indicators like heart rate and blood pressure. In addition, it is crucial to have warning systems in place along with emergency response mechanisms [5,37,38,39,40], such as wearable sensors for continuous sweat biomarker monitoring [41]. Moreover, there are other engineering approaches available to manage the effects of heat stress, including smart shirts with Bluetooth, employing RFID-based ultra-high frequency pollution detecting devices, cooling vests, newly designed construction uniforms or anti-heat-stress uniforms, respiratory rate measurements by microwave doppler radars, cutting-edge training technologies like virtual reality, the global “Hothaps” program, designing resistant buildings with features like insulated walls and roofs, air conditioning and ventilation for indoor and air movement facilities for workers who operate outside [7], and installing cooling [28] or blowing fans [24].

Administrative strategies consist of providing shade or cold rest places and cold water; having, adjusting, and optimizing work–rest schedules and recovery time [13,24,42]; implementing acclimatization plans; conducting training programs to increase workers’ knowledge, perception, and awareness of HRI and control methods [28,43,44]; risk assessment plans [7]; implementing and improving guidelines; stopping work in extreme temperatures (>40 °C) or at specific hot times of the day; selecting health criteria when recruiting workers; providing toilet facilities [7]; providing personal water bottles; and continuous medical, mental, and physiological monitoring [10,18,45,46]. In addition, motivating employees to follow the control guidelines; mindful recognition and response to early symptoms; using proper tools [28], PPE, and work uniform with low thermal insulation and high vapor permeability; extending lunchtime breaks with possibilities for taking naps; balancing between protection and production logic; management of chronic diseases; attending to elderly and susceptible staff; and implementing different protection rules for different workers [18] are other strategies. Additional management approaches to controlling heat stress in construction companies and projects include rescheduling non-essential work, observing and supervising workers with supervisors and educated coworkers, using loose-fitting uniforms or cooling vests, and enforcing mandatory daytime breaks in the summer.

Moreover, PPE is crucial in mitigating heat stress and HRIs for workers in high-risk environments. PPE can significantly reduce physical strain and metabolic heat by providing protective gear designed to minimize heat exposure. For instance, wearing lightweight, breathable fabrics and UV-protective clothing can reduce the heat absorbed from solar radiation [28]. Anti-heat-stress clothing, including cooling vests and moisture-wicking fabrics, helps regulate body temperature by enhancing sweat evaporation and reducing heat retention [7]. Furthermore, helmets with ventilation systems and cooling inserts can alleviate heat buildup, particularly for outdoor workers under direct sunlight. In addition, wearable sensing devices that monitor physiological responses such as heart rate and skin temperature allow for real-time assessment of heat stress risks, helping workers and supervisors respond proactively [39]. These innovations, combined with auxiliary cooling methods like cooling towels or ice vests, enhance the body’s ability to manage heat, reducing the likelihood of heat-related illnesses. However, it is essential to balance PPE effectiveness with comfort, as overly restrictive gear can impede heat dissipation, emphasizing the need for well-designed, purpose-built PPE in managing occupational heat stress [9].

Synthesizing all strategies and identifying overlaps and distinctions between them will provide a comprehensive understanding of how they can mitigate the diverse heat stress risk factors. Moreover, providing a structured overview of heat stress control strategies, associated risk factors, and research gaps will offer valuable insights into the interactions between the risks and related interventions. It will also guide future research and practical improvements to address these risk factors based on environmental, occupational, societal, and personal conditions.

## 3. Research Method

**Figure 1:**
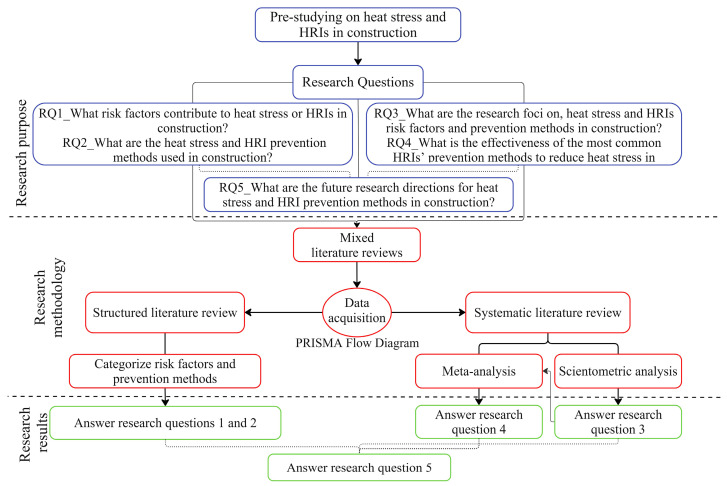
Research process and steps.

This study applies the mixed literature review methodology, involving a structured and systematic literature review to develop a comprehensive analysis of heat stress and HRIs, their risk factors, research focuses, control methods, and their effectiveness in construction. First, a structured literature review of heat stress (or HRI) risk factors is conducted, in which the risk factors are categorized into eight different groups. Heat stress (or HRI) control methods are reviewed and classified into three categories. Second, a systematic literature review uses a scientometric analysis of selected studies to elaborate on research focuses, most frequently cited risks, and preventive measures. Furthermore, a meta-analysis evaluates the effectiveness of the most recognized critical control methods. Figure 1 illustrates this study’s research process to clarify the steps and procedures.

### 3.1. Data Collection

This study considered three database sources to prevent biased results for searching and gathering the associated publications for research purposes: Web of Science, Scopus, and Engineering Index. Based on the research questions in this study, several keywords and search combinations were selected, as shown in Table 1. The search within the databases was explicitly focused on extracting keywords exclusively from publication titles. The conjunction “and” combined terms to broaden the search for more relevant publications.

A total of 1142 publications were obtained from the initial search (272 from Web of Science, 420 from Scopus, and 450 from EI). These databases were chosen due to their extensive coverage of high-quality, peer-reviewed engineering, occupational safety, and construction research literature. Web of Science and Scopus are widely recognized for their comprehensive indexing of multidisciplinary academic publications. In addition, EI (Engineering Index) focuses explicitly on engineering and technology-related research, making it particularly relevant for identifying studies related to heat stress and construction safety. After removing duplications, 755 records were reviewed based on titles, abstracts, and full texts. A total of 636 irrelevant publications were removed after reviewing the titles, keywords, and abstracts. Four eligibility or inclusion criteria were defined to examine the abstracts and full texts and exclude ineligible publications in this final synthesis. These inclusion/exclusion criteria were (a) written in the English language (full-text availability); (b) regarding the construction industry; (c) related to heat stress or heat-related illnesses; and (d) published in or after the year 2000. Excluding older publications ensured that the information gathered was more relevant to the current state of heat-related knowledge. Also, newer publications are often subject to more rigorous peer review processes and quality measures.

**Figure 2:**
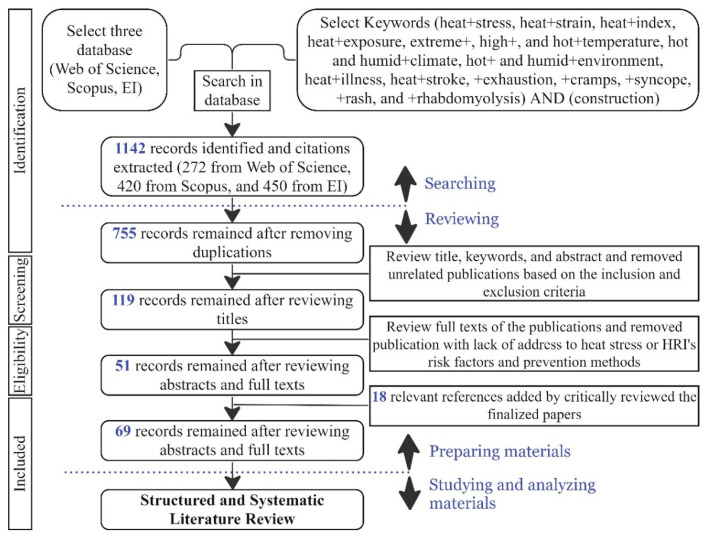
Searching procedures.

Regarding the aforementioned inclusion criteria, some publications were removed due to the full text being unavailable; the remaining were downloaded to review their full text. Complete manuscripts were critically reviewed, and the identified and irrelevant publications that did not address heat stress or HRI risk factors and control methods were excluded. Finally, 51 publications were chosen. While studying them, relevant and eligible references were downloaded and reviewed, and finally 18 relevant publications were added to the data source. Therefore, 69 full-text papers that met the inclusion criteria and aligned with the research objectives were collected and organized to conduct structured and systematic literature reviews. The data collection procedures based on the preferred reporting items for systematic reviews and meta-analyses (PRISMA) flow diagram are depicted in Figure 2. Note that further inclusion criteria were applied to select publications for the meta-analysis.

### 3.2. Scientometric Analysis

The scientometric analysis illustrates the research foci of the final 69 extracted publications to address the third research question. To understand the scientific citations and identify the research foci of the retrieved and eligible publications, this study utilizes the CiteSpace software, version 6.2.R2, developed by Chaomei Chen at Drexel University, Philadelphia, USA, for conducting the scientometric analysis [47]. According to the characteristics of the imported data that were extracted from Web of Science and Scopus, 1 paper was removed, and 68 publications from those databases were involved in the analysis. Bibliographic data of the publications were extracted in plain text format from the two databases to import into the software. A 1-year time scaling was chosen to sort publications annually. Cited references and authors were selected for the node types in the cluster analysis of co-occurring keywords to reveal the clusters of main topics and references. Also, the static cluster view was chosen as the visualization style. A structural visualization analysis was conducted, and the results are discussed further. This evaluation is beneficial in finding the most significant risk factors and cited control methods, and is also utilized as a source to identify the details of the last research question (RQ4).

### 3.3. Meta-Analysis

To quantitatively assess the effectiveness of heat stress and HRI control methods, this study conducted a meta-analysis of data retrieved from the publications focusing on the control options. A meta-analysis is a statistical way to estimate the mean and variance of population effects from a group of studies addressing the same research question [48]. Before performing the meta-analysis, three steps were processed. First, in addition to the eligibility factors applied in the search part, three more inclusion criteria were used to retrieve the final articles included in this analysis. These criteria selected articles that (a) investigated practical control methods, (b) reported a usable effect size, and (c) used different datasets. Second, according to the research question and reviews, one critical variable was chosen for the effect size measurement. This was to evaluate the continuous distribution of measurements in several studies based on the two groups’ comparisons. The effect size is a standardized measure of the observed effect size in each study or dataset [48]. Third, the effect size and sampling or pooled variance were selected for the analysis methodology. The standardized mean effect was utilized to compare two groups that used (the case group) and did not use (the control group) the specific control method. The effect size measures those groups’ standardized difference and the strength relationship between the means. Equations (1) and (2) were used to calculate and standardize the effect sizes and pooled variances [48].

(1) $$d=\frac{M2-M1}{\sigma },$$

(2) $$\sigma 2=\frac{\left(n1-1\right)s12-\left(n2-1\right)s22}{\left(n1+n2\right)-1},$$

where *d* represents the effect size, *M*1 and *M*2 represent the core body temperature means for the control and case groups, *n*1 and *n*2 represent the control and case sample sizes, *s*1 and *s*2 represent the standard deviations of the control and case groups, and *σ* represents the pooled standard deviation of the groups.

## 4. Results and Discussion

### 4.1. Publication Trend Analysis

**Figure 3:**
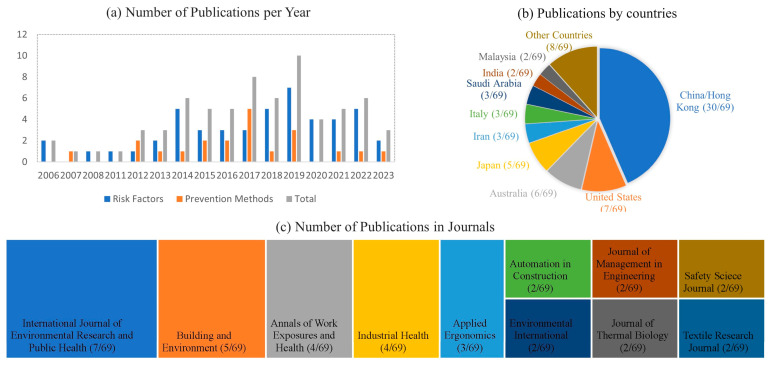
Results of publication trend analysis: (**a**) number of publications per year; (**b**) publications by country; (**c**) number of publications per journal.

The publication trend of the articles was investigated, and the results are shown in Figure 3 in three parts. Figure 3a illustrates the number of publications each year, and their research focuses on the heat stress risk factors or the prevention methods. It can be seen that 2019 and 2017 had the highest number of publications in which research studies focused on risk factors, with more in 2019 than in 2017, which focused more on prevention methods. A concentration of publications is visible in the period between 2014 and 2019. Figure 3b shows another noticeable concentration of publications based on countries, in which 43% of the publications were from China/Hong Kong, followed by the United States (10%), Australia (9%), and Japan (7%). Figure 3c, which depicts the number of articles published in different journals, indicates that the International Journal of Environmental Research and Public Health has published more than 10% of the articles included in this study. These trend analyses could help generate an overall vision of heat stress, strain, and illnesses, and characteristics of the research that has been conducted.

Many research studies evaluated and discussed different types of heat stress (or HRI) risk factors and prevention methods; these have been reviewed. The following section discusses the research foci through a scientometric analysis.

### 4.2. Scientometric Analysis Results

**Figure 4:**
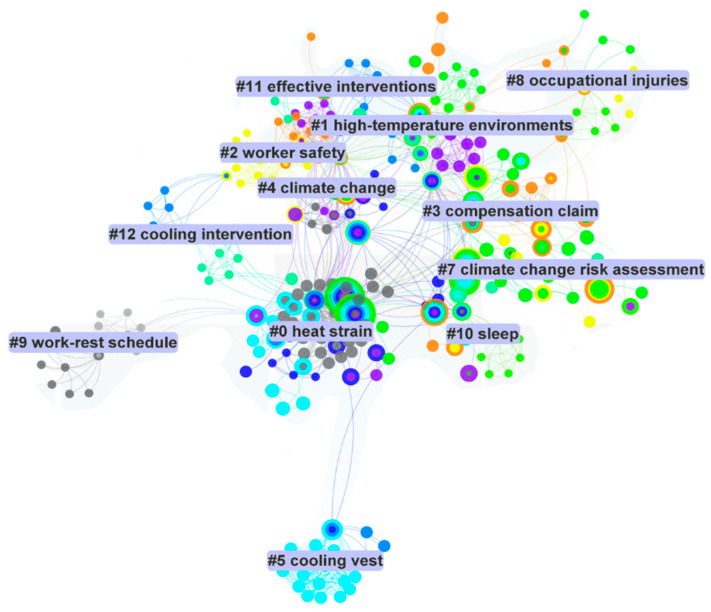
Clusters with indexing terms.

To identify the research focus and scientific references on risk factors and prevention methods, a scientometric analysis was conducted using the CiteSpace software. CiteSpace was selected for its robust capabilities in visualizing trends, mapping co-citation networks, and identifying key research themes in large datasets, making it particularly suited for this study. A network system visualizes the issue’s main topics as the nodes and their index references as the connections. The visualization results in Figure 4 illustrate that heat strain, high-temperature environments, and worker safety are the most cited critical terms. In addition, cooling vests, work–rest schedules, and cooling interventions were identified as the most significant research focus on prevention strategies. In addition, high-temperature environment was recognized as the most frequent term in heat stress risk factors.

Moreover, the central cluster, labeled “heat strain”, is a focal point, indicating its prominence as a core topic with numerous interlinked nodes, suggesting extensive research interest and foundational significance. Surrounding clusters, such as “high-temperature environments”, “worker safety”, and “climate change”, reflect interconnected research areas that directly relate to heat stress, showing how environmental and safety factors contribute to heat strain in occupational settings. The network map also highlights peripheral yet relevant topics like “cooling vest”, “work-rest schedule”, and “cooling intervention”, which are engineering and administrative controls for mitigating heat-related health risks. The presence of clusters such as “occupational injuries”, “compensation claim”, and “effective interventions” illustrates the broader implications of heat stress in terms of health outcomes, economic impact, and prevention strategies. Although this analysis provides a general overview of the most critical terms used in the reviewed publications, the in-depth assessment of the groups of risk factors and control strategies and synthesizing and categorizing them is crucial for a comprehensive knowledge of heat stress hazards and prevention methods in construction.

### 4.3. Synthesizing the Heat Stress Risk Factors

**Figure 5:**
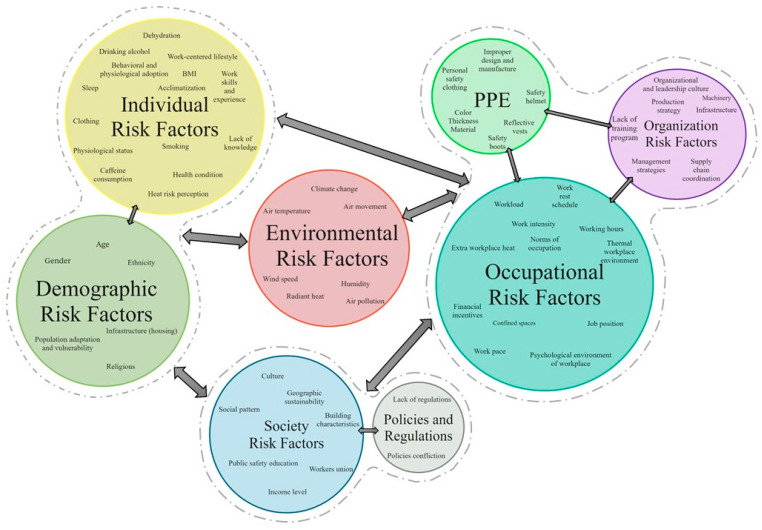
Heat stress risk factors.

Heat stress in construction is influenced by various risk factors across individual, environmental, societal, and occupational dimensions. Understanding these factors and their interactions is essential for effectively assessing and mitigating the risks associated with HRI. Rowlinson and Jia categorized these risk factors into eight levels, from personal to global levels, involving individuals, job units, teams, projects, organizations, industry, society, and the ecosystem [9]. Although these levels address several risk factors, more synthesis of additional factors needs to be explored in some research studies. Therefore, this study critically reviewed selected publications and their references and synthesized and organized all heat stress and HRI risk factors into eight groups. Figure 5 illustrates the eight groups of heat stress risk factors, categorized into four main clusters: individual and demographic factors, environmental factors, society and policies, and occupational, organizational, and PPE factors. It also shows interconnection arrows, representing the complex interactions and influences between these groups and clusters on overall heat stress vulnerability.

Cluster 1 combines individual and demographic factors, focusing on personal characteristics and demographic aspects that affect how susceptible individuals are to heat stress. Cluster 2 focuses on environmental factors, including climate, air temperature, and humidity, directly impacting individual susceptibility and occupational conditions. Cluster 3 groups society and policy factors influencing heat stress through cultural norms, public safety education, and regulatory frameworks. It shapes how societies and regulations can affect heat hazards and safety practices in construction. Cluster 4 merges occupational, organizational, and PPE factors to emphasize workplace-related risks such as work intensity, management strategies, and the role of protective equipment. The arrows demonstrate the interactions between clusters, highlighting critical connections: Environmental factors influence individual and occupational risks, as weather conditions impact both personal and job-related heat stress. Society and policies connect with individual and organizational risks, as societal norms and regulations affect personal vulnerability and workplace safety practices. These connections illustrate a dynamic feedback loop where each cluster impacts and is impacted by the others. The interconnected arrows reflect cascading effects, where changes in one cluster (e.g., stricter regulations) can influence another (e.g., workplace practices and personal vulnerability), emphasizing interdependency. Additionally, the diagram underscores the importance of considering overlapping influences to develop holistic and sustainable heat stress management strategies. This emphasizes the need for an integrated approach to managing heat stress that simultaneously addresses environmental, personal, societal, and workplace factors.

The factors influencing heat stress in construction can be categorized into eight groups: environmental, individual, demographic, occupational, organizational, PPE-related, societal, and regulatory. Each group contains distinct risk factors contributing to heat stress in construction environments. The following sections provide a detailed explanation of these risk factors within each category.

#### 4.3.1. Environmental Risk Factors

Key environmental risk factors in construction include local weather conditions such as air temperature [2,49,50], humidity [51], wind speed, and solar radiant heat [7,52]. Additional factors include radiant heat exposure [22,45], climate change [6,27], and air pollution [20], which all impact heat stress for outdoor construction workers. Individual risk factors.

#### 4.3.2. Individual Risk Factors

Individual risk factors for heat stress in construction workers include physiological and lifestyle characteristics that increase vulnerability to HRI. Key physiological factors include heart rate, blood pressure, blood sugar levels, oxygen consumption, hydration status, energy expenditure, and metabolic equivalent, which collectively help quantify physical strain in hot environments [19,20,53,54]. Personal characteristics such as body mass index (BMI), body weight, and body composition also play a role, with a higher BMI often linked to a greater risk of heat disorders [49,55]. Lifestyle factors, including physical and mental health conditions, previous HRI history, alcohol and smoking habits, acclimatization status, clothing choices, sleep quality, psychological stress, food and caffeine intake, and work-centered lifestyles, can impact a worker’s tolerance to heat stress [9,13]. Other influences include heat risk perception, knowledge, skills, and experience levels, affecting how workers respond to heat and whether they engage in protective behaviors [28,35].

#### 4.3.3. Demographic Risk Factors

Demographic factors impacting heat stress risk in construction include age, gender, ethnicity, vulnerability, housing type, cultural practices, religion, migration status, language, and adaptation levels [1,30,56]. Older workers are especially vulnerable, with age linked to more severe heat-related symptoms [57]. Ethnicity also plays a role, as Hispanic workers have been found to experience more heat-related disorders than non-Hispanics [49]. Migrant workers, who often lack acclimatization and language skills, face increased risks, compounded by limited access to heat safety training [58]. Cultural factors, such as religious clothing practices, may also reduce heat dissipation, raising risk levels for women.

#### 4.3.4. Occupational Risk Factors

Occupational risk factors for heat stress in construction include high work intensity, workload, prolonged working hours, inadequate work–rest schedules, and financial incentives that can lead to improper breaks [5,9,24]. Workers involved in outdoor manual labor in construction, common among subcontracted and project-based roles, are particularly vulnerable to HRI [59]. Job types such as steelwork, piling, roofing, crane operation, and positions involving direct contact with hot surfaces or machinery require enhanced thermal protection due to increased exposure to radiant and artificial heat sources like infrared radiation [14]. Additionally, concreting, welding, and road work may expose workers to extra environmental heat, further exacerbated by confined or poorly ventilated spaces [9,24].

#### 4.3.5. Organizational Risk Factors

Organizational-related factors influencing heat stress in construction stem from inadequate drinking water, cooling, and sanitation facilities, which many workers report dissatisfaction with yet tolerate due to economic vulnerability [28,60]. Distrust between workers and employers can hinder heat stress safety efforts, as can an organizational culture focused more on compliance than proactive safety measures [12,61]. Leadership plays a crucial role, as resource allocation, training programs, and acclimatization support directly influence heat risk management [9,62]. Practical strategies, such as education programs on heat illness symptoms and prevention, can significantly improve worker knowledge and preventive behaviors [28,63]. However, resistance to adopting innovative safety technologies limits the effectiveness of preventive measures [64]. Organizational prioritization of safety over profitability through engineering controls, prefabrication, and sustainable safety measures can substantially reduce heat stress risk [9,65].

#### 4.3.6. PPE Risk Factors

PPE-related risk factors for heat stress in construction arise from the materials and design of safety gear, which often obstruct heat dissipation. Items like safety helmets without ventilation holes, boots, and reflective vests are typically made from low-vapor-permeability and high-thermal-insulation materials, which can increase workers’ heat strain [52,66]. Reflective vests, in particular, are frequently water-impermeable, leading to discomfort and symptoms like headaches and dizziness, prompting some workers to avoid wearing them in hot weather [9]. Traditional protective clothing, with low moisture permeability and high insulation, further inhibits sweat evaporation, raising core and skin temperatures and causing excessive sweating [27]. While long-sleeved shirts offer sun protection, they can feel excessively hot during summer [29].

#### 4.3.7. Societal Risk Factors

Societal risk factors for heat stress in construction include social patterns, cultural norms, union presence, geographic susceptibility, public safety education, and income level [9,27]. Workers in subtropical and tropical climates face heightened heat stress risk due to high temperatures and humidity, particularly in low- and middle-income countries, where informal work sectors are prevalent, and climate change intensifies heat exposure [27,67]. Cultural norms can also impact risk; for example, in India, women often wear polyester over saris for modesty, which restricts heat dissipation [27]. Additionally, workers may downplay heat risks and overestimate their resilience in cultures with a strong masculine identity in construction [59,68]. Limited union presence reduces support for heat safety measures, and economic recessions can increase job discontinuity, disrupt acclimatization, and raise heat stress vulnerability [9].

#### 4.3.8. Policies and Regulations Risk Factors

Policy and regulatory risk factors for heat stress management in construction include the varying effectiveness of guidelines, standards, preventive interventions, public and industry education, and mandated rest breaks [30,59]. While many countries implement policies to address heat stress, such as standards and codes of practice in Japan, Europe, and the USA, these measures often need more employer monitoring and enforcement [69,70]. Furthermore, international standards may not fit developing countries’ needs, as physiological, cultural, and workplace differences can render ISO standards unrealistic [27,66]. In some regions, union guidelines and policies mandate work stoppages during peak heat hours, as in Oman, Hong Kong, and Dubai, yet conflicts and gaps in policy persist. Assessing the effectiveness and adaptability of heat stress prevention policies remains essential to improve safety in high-risk construction environments [9,55].

### 4.4. Synthesizing Heat Stress Control Methods

**Figure 6:**
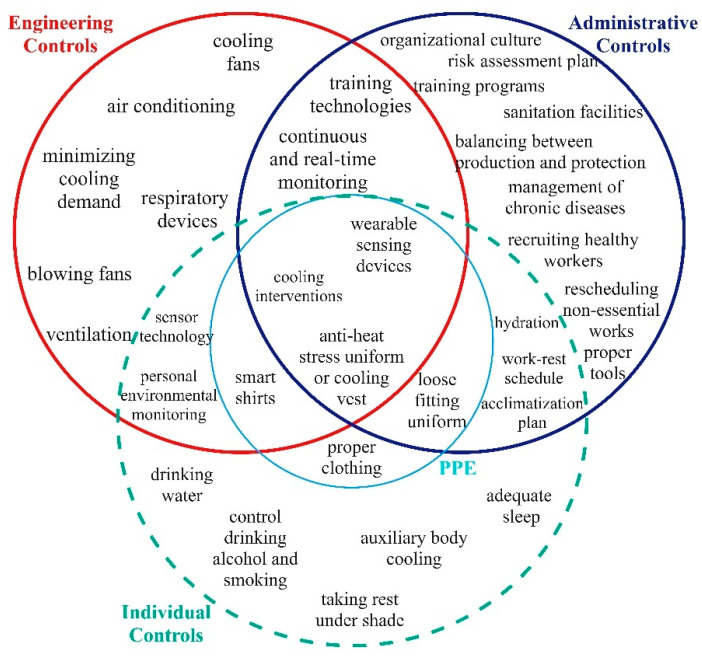
Heat stress control methods.

To effectively manage heat stress, employing a range of control strategies that address external environmental factors and individual behaviors is crucial. This study provides a comprehensive overview, as illustrated in the Venn diagram, which categorizes heat stress control strategies into engineering controls, administrative controls, PPE, and an additional layer of individual controls. Figure 6 illustrates this diagram, representing the control methods and highlighting their overlaps and distinctions. While engineering methods, administrative controls, and PPE are the traditional levels of control, this study introduces an additional barrier for managing heat stress: individual controls. Heat stress and HRI control methods that workers should perform are defined as individual control methods, including proper hydration by drinking water regularly and in a specific amount [24], acclimatization by taking a proper work–rest schedule, resting under a tent or shaded or cooled area [19,34,42,71], wearing proper clothing with a single layer and light color, wearing anti-heat-stress clothing and UV protection, using appropriate PPE to decrease the physical intensity of the work and metabolic heat [28], monitoring risk factors by wearing wearable sensing devices [39], having auxiliary body cooling, self-monitoring by using physiological responses, getting adequate sleep, and controlling destructive habits like drinking alcohol and smoking [8,14,72].

These control methods are sometimes specifically requested by workers but often overlap with other control methods. For example, hydration, work–rest schedules, and acclimatization plans are shared between individual and administrative controls, necessitating cooperation between employers and employees. Similarly, some methods span both engineering and personal levels. Notably, wearable sensing devices, cooling interventions, and anti-heat-stress uniforms or cooling vests are applicable across all levels of control [17,37,39,40,73]. Although these methods appear more practical, their implementation requires a robust safety management system to be effective.

This Venn diagram categorizes heat stress control strategies into three primary types: engineering controls, administrative controls, and individual controls, with PPE overlapping. Engineering controls include technical interventions such as air conditioning, cooling fans, ventilation, and respiratory devices to minimize the heat in the environment. Administrative controls focus on organizational practices like training programs, risk assessment plans, rescheduling work, and hydration strategies. The unique contribution of this study is the addition of individual controls, which emphasize personal behavior modifications. These personal strategies act as an additional barrier to heat stress and complement engineering and administrative solutions by focusing on individual responsibility and self-care in mitigating heat stress risks.

The overlapping sections highlight strategies that integrate elements of multiple controls. For example, continuous and real-time monitoring, cooling interventions, and wearable sensing devices combine engineering and administrative approaches by leveraging technology for worker safety and organizational oversight. The overlap of PPE across all three categories demonstrates its role in mitigating heat stress by incorporating proper clothing, cooling vests, and uniforms that facilitate heat management, contributing to personal comfort while integrating technical and organizational aspects.

### 4.5. Heat Stress Risk Factors, Control Methods, and Research Gaps in Construction

The study reviewed the literature, which is summarized in Table 2. This table presents an overview of risk factors contributing to heat stress, existing preventive measures, areas where further research is needed for control approaches, and future avenues for research studies. Table 2 categorizes various heat stress control strategies and highlights critical research gaps that warrant further exploration to enhance worker safety in high-heat environments. Engineering controls like air conditioning, ventilation, and cooling fans aim to regulate ambient temperatures, but gaps remain in optimizing fan placement and creating energy-efficient cooling systems for outdoor use. Administrative controls such as work–rest schedules, risk management plans, and training programs address workplace practices. Yet, research is needed to refine work–rest ratios specific to job roles, assess the effectiveness of risk management across industries, and examine the long-term impact of training on worker behavior and heat illness reduction. For PPE, advancements in lightweight, breathable designs, and nanotechnology-based anti-heat-stress uniforms are needed to improve comfort and efficiency, especially for high-heat environments. Personal controls, like hydration plans and cooling vests, present gaps in developing real-time monitoring solutions and understanding the durability and effectiveness of cooling vests under varying climatic conditions and physical exertion levels. Finally, wearable sensing devices for real-time heat monitoring offer promising applications, but further research is needed to integrate these with mobile alerts for better risk management.

### 4.6. Heat Stress Control Management Framework

**Figure 7:**
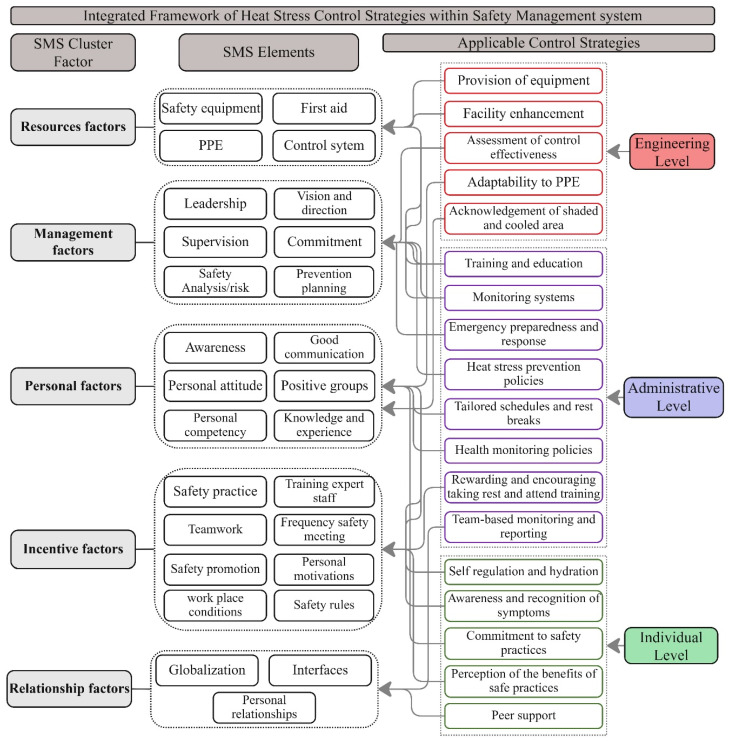
Integrated framework of heat stress control strategies within the safety management system.

Having a framework for heat stress control is crucial for systematically managing the risks associated with extreme temperatures and ensuring employee well-being. A safety management system (SMS) is a foundational resource valued for its structured approach that includes policy development, risk assessment, control measures implementation, training, effective communication, ongoing monitoring, thorough incident investigation, emergency preparedness, meticulous documentation, and continual improvement, supported by essential factors such as safety equipment, leadership, communication effectiveness, personal competency, and workplace conditions [84]. This study developed a comprehensive heat stress control framework using SMS components. Figure 7 shows this framework, the clusters of SMS safety factors, their elements and relations, the integration of the control levels in the system, and their resource allocations to prevent heat stress in construction.

This framework systematically integrates heat stress control strategies within an SMS to manage and mitigate heat-related risks in construction environments. It organizes SMS into five core clusters: Resources, management, personal, incentive, and relationship. These clusters are linked to engineering, administrative, and individual control strategies to establish a comprehensive and multi-layered approach to manage and prevent heat stress in construction. Resource factors provide the foundation for engineering controls by supplying essential equipment, like cooling vests and wearable sensing devices, and enabling facility enhancements, such as shaded areas and hydration stations. Moreover, assessing the effectiveness and adaptability of these resources to specific job positions is another method to integrate with resource factors in this heat stress management system. Management factors connect primarily with administrative controls, which set the framework for safety through policies on rest schedules, heat stress education, and monitoring systems. Leadership within this cluster supports ongoing risk assessment, emergency preparedness, and supervision, ensuring that administrative controls are effectively implemented and adapted to site conditions. Personal factors are linked closely to individual controls, equipping workers with the knowledge and competency needed for personal heat risk management, including hydration practices, symptom recognition, and self-regulation. These controls empower workers to act proactively, complementing administrative policies on worker health and acclimatization. Incentive factors encourage adherence to administrative and individual controls by fostering a safety culture that rewards proactive participation in heat management practices, such as compliance with rest breaks, training sessions, and peer monitoring. Relationship factors reinforce these controls through collaborative and communicative support networks, facilitating team-based monitoring and open feedback channels for heat safety. This resource allocation directly impacts the implementation of heat stress control strategies by ensuring the availability of necessary equipment, facilities, and systems, such as PPE, shaded areas, and monitoring tools. Adequate resource allocation supports engineering and administrative controls, enabling effective integration into workplace safety practices and ensuring sustained functionality. This integrated framework ensures that heat stress management is embedded at every level, organizationally through resources and policies and individually through personal responsibility and peer support, creating a unified, adaptive system for effective heat stress prevention on construction job sites.

### 4.7. Meta-Analysis Results

The results of the integrated structured literature review and the scientometric analysis indicated that the most popular heat stress (or HRI) control methods are using cooling vests, implementing a work–rest schedule, and applying cooling interventions. Due to insufficient datasets or quantitative summaries in the reviewed articles that evaluated the impacts or effects of work–rest schedules and cooling interventions or hydration, only the effectiveness of cooling vests had adequate datasets and was assessed using the meta-analysis. Moreover, the structured literature review illustrated that using anti-heat-stress uniforms and cooling vests is the most critical control method. At the same time, the results of the scientometric analysis also determined that using cooling vests was the most cited and primary research focus among different control strategies. Thus, the effectiveness of this method in reducing and preventing heat stress (or HRI) needed to be assessed.

This method can be in the form of uniforms, short-sleeved shirts, and vests produced with UV-protected fabric and superior heat-/moisture-transporting materials. They should be ergonomically designed, considering mobility, convenience, and safety. The research results illustrated that they could reduce core body temperature, resulting in mitigating heat stress and improving work performance and productivity [17,79,85]. Moreover, thermal and wetness sensations and heart rate can be reduced using uniforms and trousers with a specific combination of polyester/cotton materials and smart sensors and software to predict physiological outcomes [17]. A cooling vest with phase-change materials and ventilation fans can be a practical engineering method for reducing heat strain through post-exercise cooling during rest breaks in a scheduled work–rest plan [86]. Zhao et al. designed a new cooling vest, compared workers’ physiological responses during passive recovery with and without cooling vests, and showed that heat strain had been alleviated by wearing them. The two-layer cooling vest incorporates a couple of ventilation fans installed on the back and eight cooling element packs or phase-change materials on the stomach, chest, and back areas [15]. In addition, wearing them periodically for 15 or 30 min during breaks in the morning and afternoon was evaluated and identified as a feasible cooling intervention in situations where limited space and working conditions prevent the placement of a blower and water reservoir [73].

This study employed a meta-analysis to answer the last research question: “RQ4: What is the effectiveness of the most common heat stress (or HRI) control methods to reduce in construction”? Since cooling vests were selected as the most common control method, the finalized question is: “Do cooling vests effectively reduce heat stress in construction workers compared to not using cooling vests on hot days”?

After conducting the three pre-steps described in the methodology, nine datasets from nine publications were identified and selected for this analysis. The variable of core body temperature, as one of the most important indices for heat stress and a parameter represented in the results of all the selected datasets, was chosen for the effect size measurement. The mean and standard deviation of core body temperature for the case and control groups were extracted, and pooled variances and effect sizes for each data set were calculated and utilized for the meta-analysis. Table 3 shows each database, including the references, calculated means and standard deviations, and pooled variances and effect size results.

The effect size and sampling variance analytical method in the meta-analysis were calculated, and the results of the random-effects model are shown in Table 4. An estimated value of 3.06 indicated the positive and medium effect size of anti-heat-stress uniforms or cooling vests in reducing core body temperature or heat stress. Based on the results calculated in Table 3, all the cooling vests have an effect size greater than 0, so cooling intervention has a positive effect.

In addition, the heterogeneity test results are illustrated in Table 5. They indicate that the heterogeneity of the nine retrieved datasets is significant (*p* < 0.001). Among the parameters obtained, I2 evaluates how much variation in the study’s effect sizes can be attributed to the heterogeneity of the fundamental effects. This parameter represents a statistic used to quantify the degree of heterogeneity, or variation, among the effect sizes of individual studies included in this analysis. Therefore, according to the results, the information obtained in these datasets is reliable (I^2^ = 99.44%).

**Figure 8:**
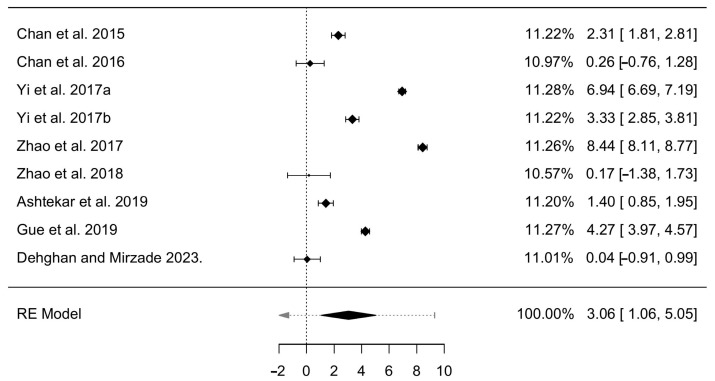
Forest plot of meta-analysis results [15,16,17,71,73,82,85,86,87].

A forest plot of the meta-analysis results is illustrated in Figure 8. The left-hand side shows the studies where datasets were retrieved, and the right-hand side demonstrates the measure of the effect for each of these studies and each study’s contributions to this analysis. Also, horizontal lines in the middle of the plot represent effect size measures and their intervals. It shows that the studies of Zhao et al., Yi et al., and Gue et al. consequently have the most significant effects on this analysis [15,16,34]. The last line also illustrates the summary measure, indicating this analysis’s estimated effect size and intervals.

Based on the text summary of the one-sided test equivalence testing, the null hypothesis test result was significant (Z = 3.008, *p* = 0.00263) and given an alpha of 0.05. Therefore, wearing cooling vests or anti-heat-stress uniforms in this meta-analysis significantly reduce core body temperature or heat stress.

## 5. Future Research Directions

The existing approaches to managing or reducing heat-related hazards were discovered by studying research papers and organizing eight categories of heat stress risk factors and three preventative measures. Table 2 outlines the areas where research regarding control methods for each risk factor could be improved. Based on these considerations, the following discussions elaborate on five avenues for further research exploration.

### 5.1. Leveraging Emerging Technologies for Monitoring and Measuring Heat Stress Risk Factors

The evaluation of strategies for preventing heat stress revealed a need for ways to measure conditions in time. Despite the use of these techniques, the current methods need to improve in this aspect. However, a potential method to address this issue is incorporating technologies like the Internet of Things (IoT), thermal imaging, and sensor technologies. IoT applications, such as wearable sensors with environmental and physiological monitoring capabilities, can provide real-time data on workers’ core body temperature, heart rate, and environmental conditions like heat and humidity. These systems can be integrated with cloud-based platforms, enabling supervisors and administrators to analyze trends, predict risks, and issue timely alerts. In addition, the IoT can support automated control systems for activating cooling interventions or adjusting work–rest schedules dynamically. Conducting research studies to assess the effectiveness and feasibility of these technologies in construction environments is essential to their successful implementation. This allows supervisors and administrators to monitor environmental and personal conditions in real time. Therefore, it is necessary to conduct research studies to assess the effectiveness of these technologies in monitoring heat stress.

Although there has been some research on using technology to monitor heat stress, a gap exists in understanding how wearable sensing devices can detect physiological and environmental data to evaluate heat-related hazards. These devices, equipped with sensors, can potentially monitor heat stress levels comprehensively. Researchers can use these technologies to assess heat stress based on personal characteristics and environmental parameters. Moreover, a potential area for research would be to evaluate types of sensors specifically designed to monitor parameters within specific body organs. This research could help identify the sensor type and determine the ideal placement within the body while considering user satisfaction and accuracy in measuring heat stress and minimizing measurement errors.

### 5.2. Impacts of Training Programs on the Knowledge of Heat Stress Control Methods

One of the risk factors highlighted in the review involves a lack of heat-related knowledge and understanding among construction workers. This can be associated with both organizational and personal practices. A practical approach to bridging this knowledge gap would be conducting heat stress training programs within the construction industry. These programs can incorporate methods and allow objective and subjective assessments of knowledge improvement. Further research can focus on evaluating the effectiveness of training programs in improving heat stress control knowledge. This can be achieved through pre-and post-training assessments, surveys to measure knowledge retention, and on-site evaluations to observe behavioral changes and compliance with safety protocols. By enhancing understanding of heat stress and HRI, associated risks, signs, symptoms, control measures, and emergency protocols, these programs would equip workers with the tools to reduce the consequences of heat injuries and illnesses. In addition, supervisors and administrators would also benefit from understanding these aspects, enabling them to reduce heat-related injuries and illnesses by providing or implementing control methods. Future studies could explore how training programs impact knowledge levels among the construction workforce, assess their effectiveness across demographics, and examine their influence on health-related behaviors.

### 5.3. Exploring the Effectiveness of Dynamic Work–Rest Schedules

Numerous scholarly articles have focused on assessing work–rest schedules in the construction industry. However, it is crucial to note that most of these studies have primarily concentrated on job roles and have often faced limitations related to working conditions. Also, more comprehensive data sets are needed to examine the effectiveness of work–rest schedules on construction job sites, especially in hot and humid environments, by performing a meta-analysis. The critical difference between a work–rest schedule and the traditional approach is the customization of schedules based on job positions. For example, a construction company or project could develop a range of work–rest schedules tailored to meet the demands of different roles. These schedules might include provisions for full-day outdoor activities, half-day outdoor tasks such as responsibilities involving driving, or infrequent outdoor work. Moreover, apart from job positions, other factors, like age, health conditions, physical status, educational background, workload intensity, and skill level, can significantly influence the effectiveness of work–rest schedules. By examining the interaction between job positions, individual characteristics, and environmental parameters, administrators and managers can contribute to refining and optimizing work–rest schedules in the construction industry.

### 5.4. Evaluation of the Effectiveness of New Cutting-Edge PPE

Several studies have examined the risk factors associated with using PPE and explored its use in preventing heat stress. Some PPE, like heat-stress uniforms, cooling vests, and loose-fitting clothing, has effectively mitigated heat-stress hazards. These strategies are measures that companies can implement to ensure the well-being of workers and prevent the adverse effects of heat stress. Alongside these garments, standard PPE like hard hats and safety boots ensure worker safety in high-temperature environments. However, this conventional PPE may need improvements, such as built-in cooling systems or the integration of materials designed to combat heat exposure, to maximize its effectiveness. Also, there is a potential research avenue in using nanotechnology to revolutionize the production of heat uniforms and vests. By leveraging nanotechnology advancements, innovative materials and designs can be developed to reduce the influence of heat radiation on the well-being and safety of workers.

### 5.5. Investigating Prospective Societal and Governmental Initiatives

The existing research on preventing heat stress in the workplace has underestimated the influence of policies and regulations. Also, there is a need to explore how sociocultural factors contribute to vulnerability to heat stress in different work settings. In addition, acknowledging that economic conditions shape susceptibility to heat stress within society is crucial. Therefore, future research should focus on understanding the interplay between culture, economy, and community vulnerability to heat stress. By examining norms and economic disparities in social contexts, researchers can understand how these factors impact workers’ perception of susceptibility to heat stress. This knowledge would be valuable for policymakers, government officials, and organizers who aim to implement strategies for preventing heat stress.

Moreover, it would be beneficial for research to assess upcoming policies and regulations regarding heat stress control strategies within the construction industry. This could involve monitoring and evaluating the development and consequences of the introduced national programs addressing heat-related hazards and control methods for outdoor and indoor environments. By examining the effectiveness of these initiatives, policymakers and governing bodies can gather insights into their efficacy and potentially identify areas that require further enhancements or adjustments.

## 6. Implications of Study to Research and Practice

This study examines heat stress and HRI risk factors among construction workers exposed to extreme temperatures, significantly contributing to construction research and practice. The study offers a comprehensive review of heat stress and HRI risk factors and control methods, eliciting critical gaps in knowledge. It also presents a systematic approach to understanding and mitigating these risks. The categorization of heat stress risk factors into eight groups, environmental, individual, demographic, occupational, organizational, PPE-related, societal, and policy-related; and control methods into three levels, engineering, administrative, and individual provides a foundational framework for researchers and practitioners to address heat stress hazards in the construction industry systematically.

This study’s findings directly impact construction practice by highlighting actionable solutions, such as the demonstrated effectiveness of cooling vests in reducing core body temperature and mitigating heat stress. These findings provide practical guidance for construction managers and site supervisors, who can implement these interventions to enhance worker safety and productivity, particularly in hot and humid conditions. Moreover, the study’s scientometric analysis identifies key research priorities and frequently cited risk factors and control strategies to equip decision-makers with evidence-based insights for better resource allocation and intervention planning. For the implications of the findings for research, the structured framework and future research directions identified in this study lay the groundwork for further investigation into heat stress prevention and worker health, fostering innovation in control strategies and policy development.

This study underscores the urgent need for evidence-based interventions and structured frameworks to ensure the safety of construction workers by bridging the gap between research and practical applications. The findings of this study could inform research and academic discourse and empower industry stakeholders with the tools and strategies needed to address the growing challenges of heat stress in the construction industry.

## 7. Conclusions and Limitations

The research objectives of this study were to undertake a thorough investigation encompassing all factors contributing to heat stress, HRI, and their corresponding control strategies, evaluate the effectiveness of the most common control methods, identify research foci and gaps, and propose future research directions. A mixed method of structured and systematic literature reviews was utilized to answer five research questions. The structured literature review answered the first couple of questions about existing heat stress risk factors and HRI control methods. Risk factors and control methods were categorized into eight and three groups, respectively. Environmental, individual, demographic, occupational, PPE, society, organizational, and policies and regulations are the eight heat stress risk factors groups. Control options were also sorted into engineering, administration, and individual (personal) groups. Cooling vests or anti-heat-stress uniforms, work–rest schedules, and hydration are each control group’s most common control options. In addition, a heat stress control framework was developed based on the safety management system for mitigating heat stress and heat-related illnesses in construction. This framework ensures a comprehensive and proactive approach by integrating engineering controls, administrative measures, and individual actions. It highlights that the collaboration between management, engineering teams, and employees fosters a safety culture crucial for effectively implementing heat stress prevention strategies.

A systematic examination of the literature encompassing scientometric analysis and meta-analysis was conducted to address the final set of research inquiries. The scientometric analysis results highlighted that the principal research foci and central themes in the realm of heat stress (or HRI), risk factors, and control strategies within the construction sector are on heat strain, high-temperature environments, the utilization of cooling vests, and implementation of a work–rest schedule. Drawing from the scientometric analysis outcomes, attention was directed toward scrutinizing the effectiveness of cooling vests in reducing core body temperature and mitigating heat stress through a meta-analysis. The findings from the meta-analysis underscore the significant effectiveness of cooling vests and anti-heat-stress uniforms in lowering core body temperature and mitigating heat stress among construction workers. The analysis of nine datasets demonstrated a positive and medium effect size for these interventions.

Considering the research gaps after reviewing risk factors and existing control methods, five opportunities for future research were proposed and discussed. Leveraging emerging technologies, such as wearable sensing devices for heat stress monitoring and measuring, conducting training programs to improve the knowledge of the construction workforce, implementing and evaluating dynamic work–rest schedules, embracing innovation and investigating novel PPEs to reduce heat stress hazards, exploring the effectiveness of sociocultural and economic determinants of heat stress vulnerability, and assessing heat stress control policies and regulations were identified as the potential directions for future studies.

There are three limitations to this study. First, the CiteSpace software used for the scientometric analysis only accepted data extracted from the Web of Science and Scopus database. These two database sources covered most of the publications included in this study, but one paper was excluded from our dataset. Second, the number of publications that studied the impacts of hydration and work–rest schedules on preventing HRI limited the ability to perform more meta-analyses to evaluate the effectiveness of these control methods. The lack of adequate and coherent datasets in the results of a few publications put another limitation on the analysis. Third, the meta-analysis was constrained by the restricted datasets and the limited number of studies based on the inclusion criteria. Therefore, researchers should interpret the meta-analysis results based on the sample sizes to conduct a more extensive meta-analysis that includes more diverse studies.

## Figures and Tables

**Table 1:** Searching keywords and combinations.

Keyword #1	Keyword #2	Keyword #3
Heat	Stress, strain, index, exposure, illness, stroke exhaustion, cramps, syncope, rash, rhabdomyolysis	Construction
Extreme, high, hot	Temperature	
Hot, humid	Climate	
Hot, humid	Environment	

**Table 2:** Heat stress risk factors, control methods, and research gaps in construction.

Control Strategy	Applicable Control Strategies (with Citations)	Risk Factor Groups Addressed	Risk Factors	Research Gaps
Engineering Controls	Air conditioning, ventilation [7,52]	Environmental	Air temperature [2,49]; humidity [51]; wind speed and direction [7]; solar radiation [27]; air quality/pollution [6,11,20,74]	Investigate energy-efficient and portable cooling solutions for outdoor worksites in high-heat environments.
	Cooling fans [28]	Environmental	Wind speed, solar radiant heat [7]; climate change-induced heat [6]; air pollution [20]	Research is needed on optimizing fan placement and airflow patterns to maximize cooling in outdoor environments. Investigate the long-term effectiveness of cooling fans in extreme conditions and their impact on overall heat strain reduction.
	Blowing fans [14,24]	Environmental	High ambient temperatures [2]; humidity [51]; climate change [27]	Study the best natural and mechanical ventilation combinations to ensure effective cooling.
	Continuous monitoring [38]	Environmental, Occupational	Workload [24,54]; high metabolic heat [8]; lack of ventilation [9]; air pollution [20]	Develop real-time heat monitoring technologies integrated with smart systems for worker safety alerts.
Administrative Controls	Work–rest schedule [13,75,76]	Occupational	Work intensity [5]; high physical demand jobs [24]; heat-related illness risks during peak hours [19]; heatstroke incidents [27,63]	Research optimal work–rest ratios tailored to specific job roles and environmental conditions.
	Risk management plans [7]	Occupational, Organizational	Prolonged heat exposure [33]; lack of safety training [9]; high-risk work environments [28]	Evaluate the effectiveness of heat stress risk management plans across different sectors and environments.
	Training programs [10,28,44]	Demographic, Occupational	Vulnerable workers [49]; migrant workers [30,77]; inexperienced workers [1]; ethnicity-specific vulnerability [49]	Examine the long-term impacts of training programs on worker awareness, behavior, and heat illness reduction.
	Recruitment of healthy workers [78]	Organizational, Demographic	Older age [1]; pre-existing health conditions [51]; alcohol consumption, smoking habits [9]; inability to acclimatize [9]	Study the recruitment practices in high-heat sectors to ensure appropriate worker health screening.
	Rescheduling non-essential works [13,34]	Occupational	Heat exposure during peak hours [19]; work–rest ratio imbalances [5]; strenuous tasks during the hottest hours [24]	Analyze the impact of rescheduling non-essential tasks on productivity and worker safety in extreme heat conditions.
	Balancing production and protection [33]	Occupational	Pressure to maintain productivity [7]; financial incentives increasing workload [9]; workload intensity [24]	Research the effects of financial incentives on workers’ safety and heat illness risk in high-heat environments.
PPE	Anti-heat-stress uniforms, cooling vests, loose-fitting uniforms [15,28,73,79]	Environmental, Occupational	Improper PPE design [29]; heat build-up in safety helmets without vent [66]; use of reflective vests [7]; excessive sweating and discomfort [27]	Research the development of more lightweight and breathable PPE that still meets safety standards for high-heat environments.
	Proper PPE [7]	Individual, Occupational	BMI-related heat risk [49]; High heart rate during work [20]; Fatigue and dehydration [9]; Clothing insulation and evaporation barriers [29]	Investigate advancements in nanotechnology to produce more efficient anti-heat-stress uniforms and gear.
Personal Controls	Hydration plans, auxiliary body cooling [46,53,80]	Individual, Occupational	Lack of hydration [24]; electrolyte imbalances [19]; dehydration-induced heatstroke [5,57]; improper fluid intake [9]	Develop personalized hydration plans using wearable sensors to monitor hydration levels in real time.
	Adequate sleep, self-monitoring using physiological responses [14,81]	Individual	Fatigue [33]; lack of adequate sleep [9]; pre-existing medical conditions [46]; alcohol consumption [9]	Explore the relationship between sleep patterns, fatigue, and heat-related illness risk in workers exposed to extreme heat.
	Cooling vests, proper clothing [15,16,82,83]	Individual, Occupational	Loose-fitting uniforms [18]; lack of UV protection [28]; health management issues [1]; poor acclimatization [9]	Research the effectiveness of cooling vests across different climatic conditions and physical exertion levels. Study cooling vests’ durability, comfort, and wearability for prolonged use in construction environments.
	Wearable sensing devices [38,39,41]	Individual	Heat risk perception [74]; lack of real-time monitoring [37]; undetected heat strain [39]	Investigate integrating wearable heat sensors with mobile apps for real-time alerts and risk management.

**Table 3:** Cooling vests’ effectiveness results.

Subject	Reference	Sample Size	Mean		SD		$\sigma^{2}$	d
			Pre	Post	Pre	Post	Variance	Effect Size
Newly designed uniform	[85]	184	38.39	38.24	0.30	0.20	0.065	2.308
Short-sleeved T-shirt	[82]	10	37.96	37.89	0.55	0.49	0.271	0.258
New T-shirt uniform	[17]	10	38.45	38.34	0.11	0.14	0.016	6.94
Cooling vest with ventilation fans	[86]	10	38.27	38.07	0.25	0.24	0.06	3.331
Cooling vest with ventilation fans	[15]	12	38.13	37.89	0.20	0.13	0.028	8.436
New anti-heat-stress uniform	[73]	14	37.82	37.71	0.87	0.71	0.63	0.175
New designed cooling vest	[71]	29	36.89	36.78	0.28	0.28	0.078	1.403
New T-shirt uniform	[16]	173	38.37	38.27	0.12	0.18	0.023	4.274
Evaporative cooling vest	[87]	30	36.37	36.36	0.55	0.41	0.235	0.043

**Table 4:** Random-effects model results.

	Estimate	SE	Z	*p*	CI Lower Bound	CI Upper Bound
Intercept	3.06	1.02	3.0008	0.00263	1.065	5.046

**Table 5:** Heterogeneity statistics results.

Tau	Tau^2^	I^2^	H^2^	R^2^	df	Q	*p*
3.022	9.1337 (SE = 4.6427)	99.44%	178.438	94%	8.000	1207.733	<0.001

## Data Availability

Some data, like the plain text databases used for scientometric analysis and meta-analysis, are available from the corresponding author based on reasonable request.
